# Haloferax mediterranei Cells as C50 Carotenoid Factories

**DOI:** 10.3390/md19020100

**Published:** 2021-02-10

**Authors:** Micaela Giani, Zaida Montero-Lobato, Inés Garbayo, Carlos Vílchez, José M. Vega, Rosa María Martínez-Espinosa

**Affiliations:** 1 Department of Agrochemistry and Biochemistry, Biochemistry and Molecular Biology division, Faculty of Science, University of Alicante, Ap. 99, 03080 Alicante, Spain; micaela.giani@ua.es (M.G.); rosa.martinez@ua.es (R.M.M.-E.); 2 Algal Biotechnology Group, CIDERTA-RENSMA and Faculty of Experimental Sciences, University of Huelva, 21007 Huelva, Spain; zaida.montero@dqcm.uhu.es (Z.M.-L.); garbayo@uhu.es (I.G.); 3 Department of Plant Biochemistry and Molecular Biology, Faculty of Chemistry, University of Seville, 41012 Seville, Spain; jmvega@us.es

**Keywords:** *Haloferax mediterranei*, bacterioruberin, carotenoids, C/N ratio, osmotic stress

## Abstract

*Haloarchaea* produce C50 carotenoids such as bacterioruberin, which are of biotechnological in-terest. This study aimed to analyze the effect of different environmental and nutritional conditions on the cellular growth and dynamics of carotenoids accumulation in *Haloferax mediterranei*. The maximum production of carotenoids (40 µg·mL^−1^) was obtained during the stationary phase of growth, probably due to nutrient-limiting conditions (one-step culture). By seven days of culture, 1 mL culture produced 22.4 mg of dry weight biomass containing 0.18 % (*w*/*w*) of carotenoids. On the other hand, carbon-deficient cultures (low C/N ratio) were observed to be optimum for C50 bacterioruberin production by *Hfx. mediterranei*, but negatively affected the growth of cells. Thus, a two-steps process was evaluated for optimum carotenoids yield. In the first step, a nutri-ent-repleted culture medium enabled the haloarchaea to produce biomass, while in the second step, the biomass was incubated under osmotic stress and in a carbon-deficient medium. Under the conditions used, the obtained biomass contained 0.27% (*w*/*w*) of carotenoids after seven days, which accounts for 58.49 µg·mL^−1^ of carotenoids for a culture with turbidity 14.0.

## 1. Introduction

Halophilic archaea or haloarchaea are extremophilic microorganisms from the domain Archaea that grow optimally in culture media with high salt concentrations (up to 4 M). The family *Haloferacaceae* in particular includes non-photosynthetic and mostly aerobic heterotrophs whose natural environments are usually hypersaline lakes and salt marshes [1]. Haloarchaea produce highly valuable products of biotechnological interest, such as high temperature-resistant enzymes [2], polyhydroxyalkanoates [3], polyhydroxybutyrate [4], and carotenoids [5]. The major carotenoids synthesized by haloarchaea are bacterioruberin (BR) and its derivatives monoanhydro- and bisanhydro-bacterioruberin [6,7]. 

BR is a C_50_ carotenoid that consists of a primary conjugated isoprenoid chain with thirteen conjugated carbon–carbon units and four hydroxyl groups arising from the terminal ends. It improves the fluidity of the cell membrane and, due to its strong antioxidant properties, protects the cells from the harmful effects of radiation, as well as from osmotic stress [8]. C_50_ carotenoids show a higher antioxidant capacity than the C_40_ carotenoids, such as β-carotene, that are produced by most photosynthetic organisms [9]. This property can be explained by the higher number of pairs of conjugated double bonds, which makes these carotenoids remarkably interesting for the food and pharmaceutical industries [5]. Among the possible applications of this compound, its potential uses in biomedicine as antitumoral, antiviral or spermatic mobility enhancers are of particular relevance [10]. Moreover, it also provides a potential strategy in the prevention of skin cancer, as it can repair damage in human DNA strands occurring due to exposure to ionizing radiation such as ultraviolet radiation (UVR) [11]. Recently, the pigments of haloarchaea have produced promising results as astronomical biosignatures, as these microorganisms can produce remotely observable blooms [12]. 

Generally, carotenoids are obtained through conventional methods, such as direct extraction from plants and downstream purification using chemical procedures. However, their production from microorganisms enables more economic and environmentally friendly procedures [13,14]. The use of extremophilic microorganisms is recommended as the conditions required for their growth prevent contamination by other microorganisms, even under non-sterile conditions, which plays a crucial role in decreasing the energy costs [15]. Moreover, haloarchaea require a high concentration of salt in the culture medium, which facilitates the extraction of carotenoids since the exposure of the cells to a low-salt environment induces membrane lysis. *Hfx. mediterranei* is a highly promising candidate for the production of carotenoids owing to its rapid growth and ability to consume a variety of carbon sources [16]. Furthermore, the physiology and metabolism of this organism have been widely studied, which makes it a highly useful model in different fields. However, there is a lack of in-depth studies in the field of C_50_ production using *Hfx. mediterranei* as a cell factory. The optimization of the conditions for carotenoid production is a key point to ameliorate economic costs. 

Since carbon and nitrogen are key elements in the structures of lipids, proteins, complex carbohydrates, and nucleic acids, variations in their availability might affect not only the accumulation of carotenoids but also the growth of the organisms. Furthermore, magnesium ions are required by haloarchaea for optimal growth and development [17]. However, none of the previous studies have investigated the effect of nutritional factors on the accumulation of carotenoids in *Hfx. mediterranei*. In this work, we expose this halophilic archaea to different culture conditions, such as various concentrations of inorganic salts, carbon sources, nitrogen, and magnesium. We also evaluate the effect of the carbon/nitrogen balance on carotenoid biosynthesis. 

## 2. Results

### 2.1. Carotenoids Content of Hfx. mediterranei All along the Growth Curve

**Figure 1:**
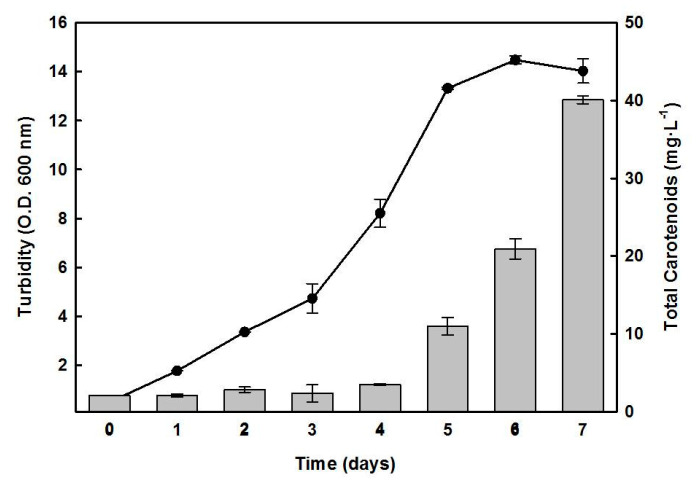
Time course of the growth of *Hfx. mediterranei* and the cellular content of carotenoids. Cells were grown in a complex medium. At the indicated times, turbidity (-●-) and carotenoids production (bars) were determined. Error bars show the standard deviation of replicates. More details are provided in the Materials and Methods section.

Figure 1 presents the typical growth curve of *Hfx. mediterranei* cultured in a complex medium, under standard conditions. Following a lag phase of three days, an exponential phase was observed during days 3 to 5, with a generation time of around 32 h for cell growth, and the stationary phase was reached after five days of growth. Figure 1 also depicts the temporal evolution of cellular carotenoids content, which increased after the fourth day of growth. The highest accumulation of carotenoids was observed during the stationary phase, when the biomass production in the culture had stopped possibly due to nutrient limitation. On day 7, the cellular carotenoid accumulation was approximately 10- to 15-fold higher than the average content of a 1- to 3-day old culture, with a yield of 40.0 µg mL^−1^. The maximum absorbance at 600 nm under these conditions was 14.0, which corresponded to 22.4 mg of biomass, containing 0.18% of carotenoids on a dry weight basis.

### 2.2. Effects of Nutrient Limitation on the Growth of Hfx. mediterranei and Synthesis of C50 Carotenoids

**Figure 2:**
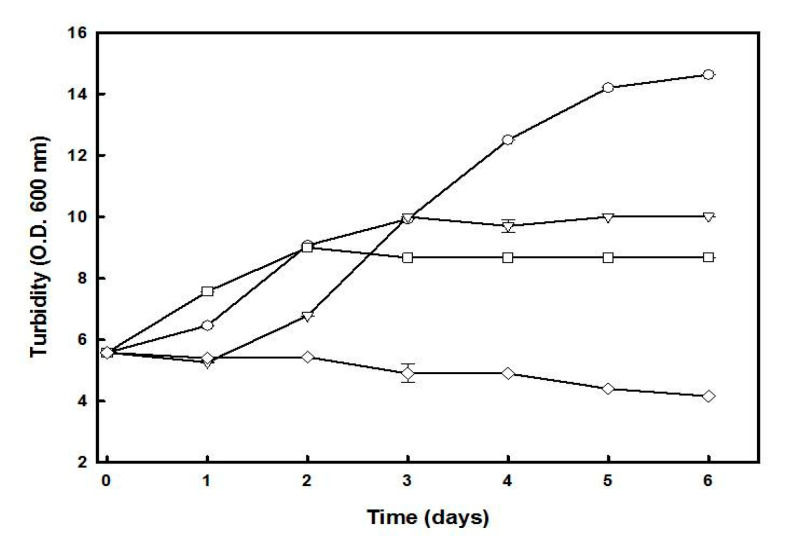
Effect of glucose and/or yeast extract starvation on the growth of *Hfx. mediterranei*. Cells growing in complex medium (OD_600_ of approximately 6.0) were harvested, washed, and resuspended in complex medium (-○-), lacking glucose (-Δ-), lacking yeast extract (-□-), or lacking both (-◇-). The turbidity was determined at the indicated times. Error bars show the standard deviation of replicates. More details are provided in the Materials and Methods section.

The effect of carbon and/or nitrogen starvation on the growth of *Hfx. mediterranei*, and the cellular accumulation of C50 carotenoids was studied using a two-step cultivation procedure. In the first step, cells were cultivated in a complex medium until the absorbance of the culture at 600 nm reached a value of about 6.0 (3.5 days). For the second step, the cells were centrifuged and resuspended in a fresh medium lacking glucose, yeast extract, or both. The culture lacking both glucose and yeast extract was unable to grow throughout the experiment (Figure 2). However, cultures lacking either glucose or yeast extract alone showed growth until the third day, which was possibly due to the mobilization of the intracellular reserves of carbon and nitrogen that supply energy and materials to support a transient maintenance of cellular functions. No significant growth was observed in these cultures from the third to the sixth day. After six days of incubation under a deficit of glucose or yeast extract, the biomass production was reduced by 40% and 35%, respectively, compared to the control culture, which reached the stationary phase at that time. 

**Figure 3:**
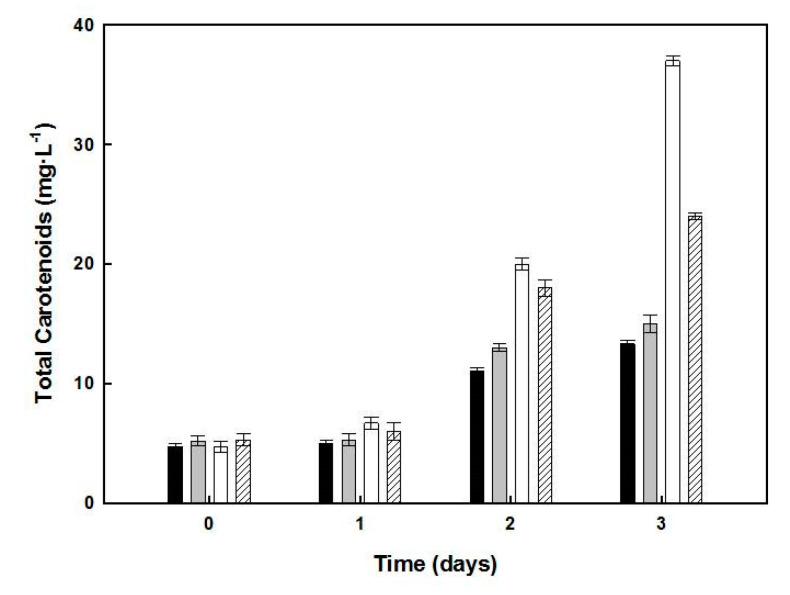
Effect of glucose and/or yeast extract starvation on the carotenoids production by *Hfx. mediterranei*. Samples of cultures corresponding to days 1, 2, and 3, under nutrient deficiency (see Figure 2), were used as the sources of carotenoids, which were extracted and quantified. The data correspond to control culture (dark bars), lacking yeast extract (grey bars), lacking glucose (white bars), or lacking both yeast extract and glucose (dashed bars). Error bars show the standard deviation of replicates. More details of experimental procedures are provided in the Materials and Methods section. This yield was obtained from a two-step procedure over seven days and was slightly lower than that obtained in the one-stage culture. Cultures lacking yeast extract showed a 1.77-fold higher carotenoid content than the control, while those lacking in both yeast extract and glucose increased the carotenoid content by 1.23-fold only.

The carotenoids content of cells from the different cultures was analyzed on days 1–3 of the second step of growth (Figure 3). In cultures lacking glucose, the cell cultures presented 37.6 µg·mL^−1^ of carotenoids content, accounting for 0.27% of the biomass in 13.92 mg dry weight. Since the absorbance at 600 nm of the second-step culture lacking added glucose was 9.0, the amount of carotenoids obtained for cultures with turbidity 14.0 (used in the one-step culture) should be around 58.49 µg·mL^−1^.

### 2.3. Effect of Inorganic Salts on the Growth and BR Production of Hfx. mediterranei

**Figure 4:**
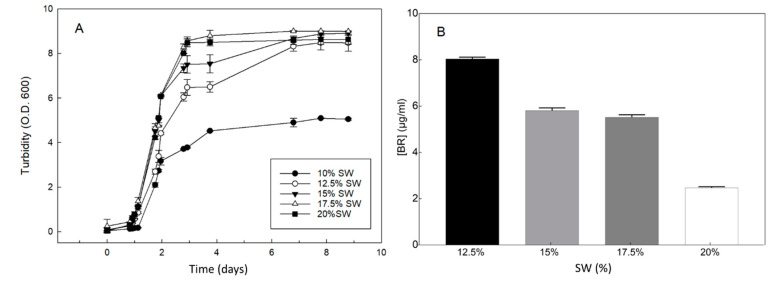
Effect of inorganic salts on the growth of *Hfx. mediterranei* and the yield of bacterioruberin (BR). The cells were grown in a complex medium, except that the concentration of inorganic salts (SW = salts in water) was as indicated. (**A**) Turbidity was measured using aliquots of each culture. (**B**) BR was measured at the stationary phase of growth (turbidity 8.0–9.0). Error bars show the standard deviation of replicates. Other conditions are described in the Materials and Methods section.

*Hfx. mediterranei* cells were grown in a complex medium containing five different concentrations of inorganic salts in the culture medium (10%, 12.5%, 15%, 17.5%, and 20%). The culture with the lowest concentration of inorganic salts, 10% (*w*/*v*), showed significantly limited growth, while the optimal growth rate was observed at 17.5% salt concentration (Figure 4A). The highest pigmentation was observed at 12.5% (*w*/*v*) salt concentration (Figure 4B). 

### 2.4. Effect of Carbon/Nitrogen (C/N) Ratio in the Medium on BR Production by Hfx. mediterranei

The *Hfx. mediterranei* cells were grown in a minimal medium (lacking yeast extract) containing nitrate as the sole N-source and glucose as the carbon source. All the cultures with KNO_3_/glucose reached an optical density of 1.8–2.5 at the stationary phase of growth, except that of 0.5 mM KNO_3_/glucose, which did not provide enough nitrate to allow normal growth (Figure S1). Table 1 shows how the cell cultures with low C/N ratio yielded the best results in terms of cell pigmentation, particularly at 15% SW. Among glucose and starch, the latter showed better results of cell pigmentation, although the C/N ratio was the same. This may have been due to the insolubility of starch which could have limited glucose availability of the cells. Table 1 also shows that the pinkish/reddish pigmentation of *Hfx. mediterranei* cultures increased with an increase in nitrate concentration in the culture medium. The pigments contained BR, as determined by its characteristic absorption spectrum, with maxima at 530, 495, and 470 nm. 

### 2.5. Effects of Magnesium or Sulfate on the Growth and Synthesis of Pigments by Hfx. mediterranei

Magnesium is a common component of microbiological media. Haloarchaea are generally considered to require more Mg^2+^ ion for growth than other microorganisms [18]. The complex medium used in this study for the growth of *Hfx. mediterranei* contained a total of 145.2 mM Mg^2+^ (64 mM from MgCl_2_·6H_2_O, plus 81.2 mM from MgSO_4_·7H_2_O). We screened the effect of a range of Mg^2+^ concentrations, from 68 to 389.0 mM, on the growth rate and carotenoid accumulation by this haloarchaea. In this experiment, the sulfate concentrations varied were from 4.1 to 325 mM. No significant differences were observed in either the growth rate or carotenoid accumulation in cells (Figure S2).

## 3. Discussion

C_50_ carotenoids are the main pigments of haloarchaea, although small amounts of C_30_, C_40_, and C_50_ carotenoids are also present [19]. Montero-Lobato et al. [7] separated and analyzed, by HPLC and spectrophotometry, the carotenoid profile of the acetone extract from *Hfx. mediterranei*. They identified bacterioruberin, monoanhidrobacterioruberin, and bisanhidrobacterioruberin as main components, comprising 89.13% of the total carotenoid content. However, previous studies on this organism have reported lower percentages of BR: 52.4% and 70% [20,21]. This variation demonstrates the influence of culture conditions on the yields of carotenoids and their composition of the haloarchaea. The BR content varies highly depending on the species of haloarchaea, with previous studies reporting contents of 98.1% in *Halorubrum* sp. [22], 68.1% in *Haloarcula japonica* [23], 49.2% in *Halobacterium* SP–2, and 55.3% in *Halorubrum* SP–4 [15]. Moreover, engineered *Hfx. mediterranei* has interestingly provided an efficient platform for high-level production of lycopene [24]. 

Induction of carotenoid accumulation by nutrient starvation has been reported in microalgal cultures, such as *Dunaliella salina* [25], *Nannochloropsis gaditana* [26], and *Haematococcus pluvialis* [27]. The one-step procedure shown in Figure 1 yielded 22.4 mg.mL^−1^ of *Hfx. mediterranei* biomass, of which 0.18% was due to carotenoids in the stationary phase of the culture, suggesting the accumulation could be triggered by nutrient limitation or low C/N ratio. To understand which nutritional conditions favored the induction of carotenogenesis in *Hfx. mediterranei*, a two-step process was performed (Figure 3 and Figure 4). The results indicated that the maximum BR yield of 33.07 μg in a biomass of 13.92 mg, with carotenoids representing 0.27% of the dry weight, was obtained in glucose-deprived cultures, provided that the yeast extract was added (low C/N ratio in the culture), indicating that this ratio played a relevant role as a possible inducer of carotenogenesis in this haloarchaea. 

Fang et al. [21] also proposed a two-stage process to produce carotenoids with *Hfx. mediterranei*. However, their yield was low, probably because, in the second step, a culture medium containing 0.1% glucose, 50 mM Tris-HCl, pH 7.2, and 5% NaCl was used. In our case, the second step was performed in complex medium, lacking added glucose, in which the C/N ratio was low. Under the conditions we used, the carotenoid yield was improved by 25-fold. Calegari-Santos et al. [17] proposed a two-step process to produce carotenoids from *Hfx. mediterranei* ATCC 33500, consisting of an initial 48-h step indicating that glucose is an inductor of carotenoid biosynthesis in this haloarchaea. However, we obtained maximum cells pigmentation in glucose-lacking cultures, which may be due to the double role of glucose, as nutrient and osmolyte, contributing to the osmotic pressure.

The data in Table 1, obtained with cultures in minimal medium, show that the C/N ratio has a direct effect on the pigmentation level of the cells. Particularly interesting is the effect exerted by the starch on the cell pigmentation when added as the carbon source to the medium instead of glucose. To the best of our knowledge, the latter observation and its potential applicability are novel aspects in this research field.

Osmotic pressure appears to be one of the main factors inducing carotenogenesis in *Hfx. mediterranei*. As shown in Figure 4B, a decrease in the inorganic salt concentration in the culture medium induced the accumulation of carotenoids in cells, although the growth rate was reduced. This finding is consistent with that of previous reports on haloarchaea [28]. We have already published a study showing that 12.03% (*w*/*v* of NaCl) in the complex medium is optimum for carotenoid production in *Hfx. mediterranei* [7]. In this study, we examined the effect of inorganic salts on growth and carotenoid biosynthesis in this haloarchaea, which mean that all the inorganic salts present in the complex medium were equally diluted. The results obtained are very similar. In this context, and probably related to the osmotic pressure, the presence of oxidative stress-inducing compounds in the culture, such as H_2_O_2_, induces pigmentation in *Hfx. mediterranei* as a mechanism to protect the cells from oxidative injury [29]. 

The response of halotolerant microorganisms to magnesium is dependent on the genus and the species. For instance, according to Soliman and Trüper [18], the presence of 10 mM Mg^2+^ in the culture medium significantly inhibited the growth of *Halobacterium pharaonic*, whereas *Halobacterium volcanii* required high magnesium concentrations (100 mM) for optimal growth [30]. Similarly, *Halobacterium sodomense* isolated from the Dead Sea also shows an optimal growth between 0.6 and 1.2 M Mg^2+^ [31]. *Hfx. mediterranei* shows a high tolerance to the presence of magnesium in the culture. We did not observe any remarkable effect of added magnesium, at concentrations as high as 1.0 M, on the growth and production of carotenoids in this haloarchaea using a complex medium (except for the Mg^2+^ concentration) (Figure S2). However, Fang et al. [21] observed that optimal pigment accumulation by *Hfx. mediterranei* was obtained in the culture medium containing 325 mM magnesium and 5% (*w*/*v*) NaCl, 0.1% glucose, and 50 mM Tris-HCl, pH 7.2. This different behavior of the haloarchaea may be due to the different culture conditions used.

According to our data, for large-scale production of BR with *Hfx mediterranei,* a two-step procedure seems to be more promising. It is possible to increase the biomass concentration in the first step, while the second step, for biomass enrichment by 0.27% (*w*/*w*) in carotenoids, can be improved in different ways in order to increase the carotenoids productivity—either by increasing biomass productivity or by triggering the biomass enrichment in carotenoids; for instance, by using starch and nitrate.

## 4. Materials and Methods

### 4.1. Microorganism and Standard Growth Conditions

The *Hfx. mediterranei* strain R-4 (ATCC33500) was used for all the experiments. The strain was cultured in a liquid medium as formulated by Fang et al. [21] containing (per liter): NaCl, 156 g; MgCl_2_·6H_2_O, 13 g; MgSO_4_·7H_2_O, 20 g; CaCl_2_·6H_2_O, 1 g; KCl, 4 g; NaHCO_3_, 0.2 g; NaBr, 0.5 g (SW = salts in water = 20% *w*/*v*); yeast extract, 5 g; glucose, 10 g, and pH 7.3 (adjusted using 15 mM Tris-HCl) (complex medium). The cultures were incubated under standard aerobic conditions at 37 °C and 100 rpm in an orbital shaker. The starter culture was prepared in 100 mL of liquid medium contained in a 250 mL flask and incubated until the exponential phase of growth was achieved. This culture was used as the source of inoculum at a concentration of 10% (*v*/*v*) in 250 mL of liquid medium in 500 mL flasks. In some parts of this study, accordingly indicated, *Hfx. mediterranei* was aerobically grown in a minimal medium, containing all the ingredients of the complex medium, except that yeast extract was lacking. 

### 4.2. Cultivation in the Absence of Glucose and/or Yeast Extracts

The experiments conducted to evaluate the growth and carotenoid accumulation by *Hfx. mediterranei* in the absence of glucose and/or yeast extract were performed in a two-stage cultivation process. In the first stage, the cells were grown in a complex culture medium until the turbidity (OD) at 600 nm reached approximately 6.0. In the second stage, cells were harvested from the cultures of the first stage by centrifugation at 1600× *g* for 45 min, resuspended in an equal volume of fresh medium without glucose and/or yeast extract, and then cultured under standard conditions. 

### 4.3. Growth Determination

Growth specific velocity (Equation (1)) and duplication time (Equation (2)) were calculated for each growth condition using the following formulae:

µ = ln (X − X_0_)/(t − t_0_) (1)

Dt = ln(2)/µ(2)

The biomass was determined by measuring the turbidity of the culture at 600 nm using a UV-VIS spectrophotometer (Evolution 201, Thermo Fisher Scientific, Watham, MA, USA). The dry weight was determined using a 1 mL sample of the corresponding culture, which was filtered through a pre-weighed membrane (ϕ = 0.2 μm), and the retained cells were washed on the filter using 5 mL of 1% (*w*/*v*) NaCl solution. The membrane was then dried at 80 °C until a constant weight was reached. A control sample of 1 mL of the uninoculated culture medium was incubated simultaneously with the culture, and its dry weight obtained was later deducted from that of the culture sample. A culture with an OD of 1.0 at 600 nm had a dry weight of 1.60 g·L^−1^.

For extraction of the carotenoids, 10 mL of the culture samples were centrifuged at 1600× *g* for 45 min, the harvested cells were lysed by freeze/thawing, and the biological material was finally suspended in 1 mL of pure acetone and kept overnight at 4 °C. The suspension was then centrifuged at 3000× *g* for 5 min and the total carotenoid content of the supernatant was determined by measuring the absorbance at 494 nm and using an extinction coefficient, ε (1%) = 2540, according to the following equation:

mg·L^−1^ = (OD494/2540) × 10^4^ (3)

The pigments in acetone extract were analyzed by thin-layer chromatography as described by Strand et al. [32]. A silica-based TLC plate (Merck 5553) was used, with 50% acetone in n-heptane (*v*/*v*) as the development liquid. The Rf value of each spot on the developed chromatogram was calculated, after which each spot was scraped off and extracted with acetone for spectrophotometric analysis. The UV-VIS (300–600 nm) absorbance spectra were determined using a spectrophotometer (Evolution 201, Thermo Fisher Scientific, Waltham, MA, USA).

### 4.4. Statistical Analysis

Unless otherwise indicated, the tables and figures show the means and standard deviations of the three independent experiments. The data groups were analyzed with a one-way analysis of variance using the SPSS version 19 statistical analysis package (IBM, New York, NY, USA). Differences were considered significant when *p* < 0.05. 

## 5. Conclusions

From the results presented in this paper, a hypothetical production process of C50 carotenoids BR from *Hfx. mediterranei* should consist of a two-step process: the first step for the continuous production of biomass under the optimal growth conditions of the haloarchaea and the second step for induction of carotenoid biosynthesis, using a complex culture medium lacking glucose (low C/N ratio) with 12.5% SW. The cells from the second step should be collected during the stationary phase of growth, and the carotenoids fraction should be immediately extracted.

## Figures and Tables

**Table 1:** Effect of the C/N ratio on growth and pigmentation level in cultures of *Hfx. Mediterranei*.

Cell Culture ^1^		C/N Ratio	Turbidity (OD 600 nm)	Pigmentation Level ^1^
SW (%)	Nitrate (mM)			
25	5	5.6	2.1	-
25	25	1.1	2.3	++
25	100	0.28	2.4	+++
15	5	5.6	1.8	+
15	25	1.1	1.9	+++
15	100	0.28	2.2	++++
15	100 (S)	0.28	2.0	+++++

^1^ The cells were grown until stationary phase (6 days) in minimum medium containing 2.8 mM glucose (0.5%), except that labeled with S, which contained starch as carbon source. SW and nitrate were used as indicated. The pigmentation level was estimated as - (absence of color) or a range from + (low level) to +++++ (high level).

## Supplementary Materials

The following are available online at https://www.mdpi.com/1660-3397/19/2/100/s1, Figure S1: Effects of different concentrations of nitrate on the growth of *Hfx. mediterranei*, Figure S2: Effects of magnesium sulfate concentration on the growth and carotenoid production of *H. mediterranei*.
