# Supplementary figures and images for: Haloferax mediterranei Cells as C50 Carotenoid Factories
Source: Mar Drugs. 2021 Feb 10;19(2):100. doi: 10.3390/md19020100 (PMC7916556; doi:10.3390/md19020100)

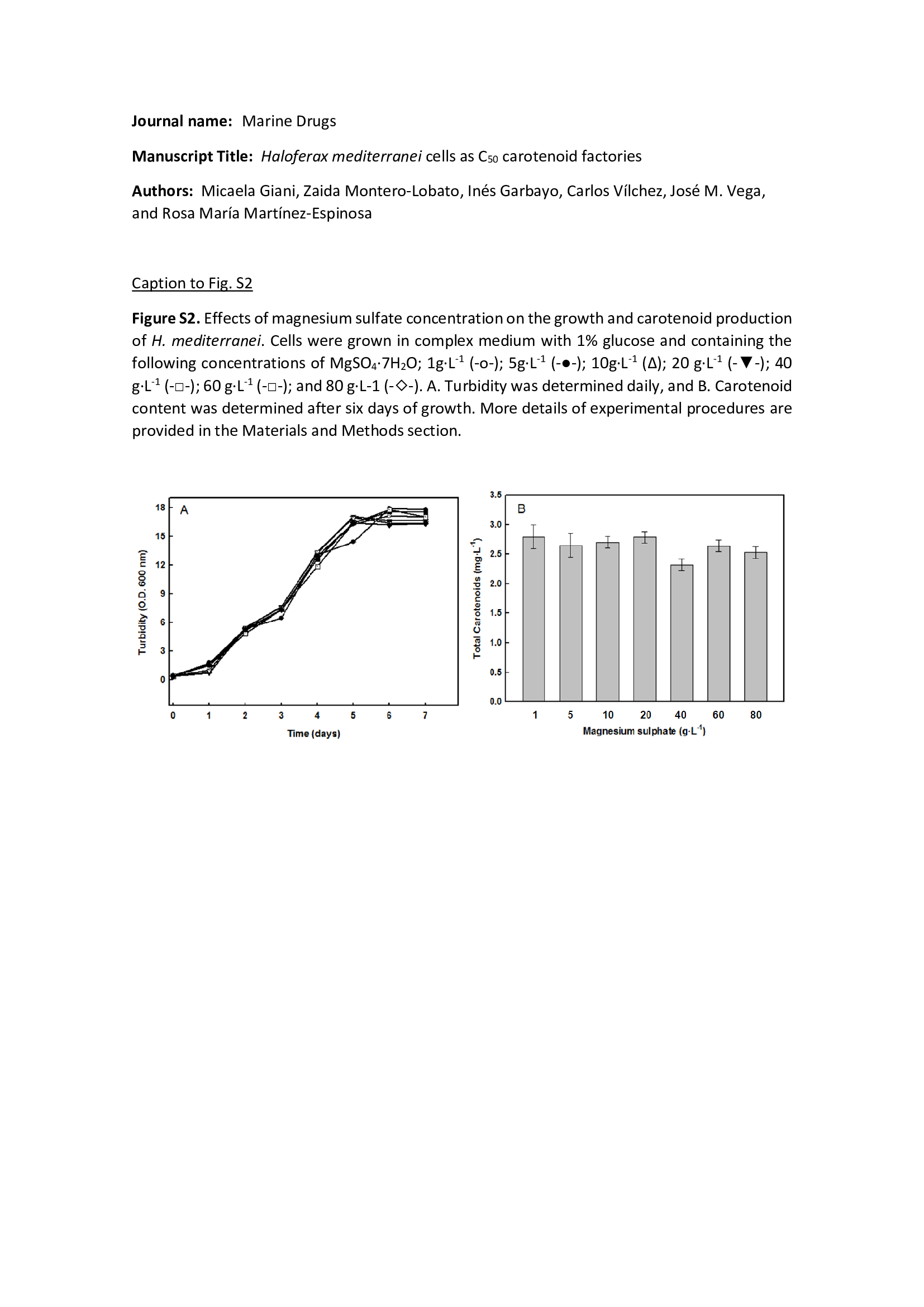

Supplement: Supplementary file 1 [file marinedrugs-19-00100-s001.zip › Fig-S2.jpg]

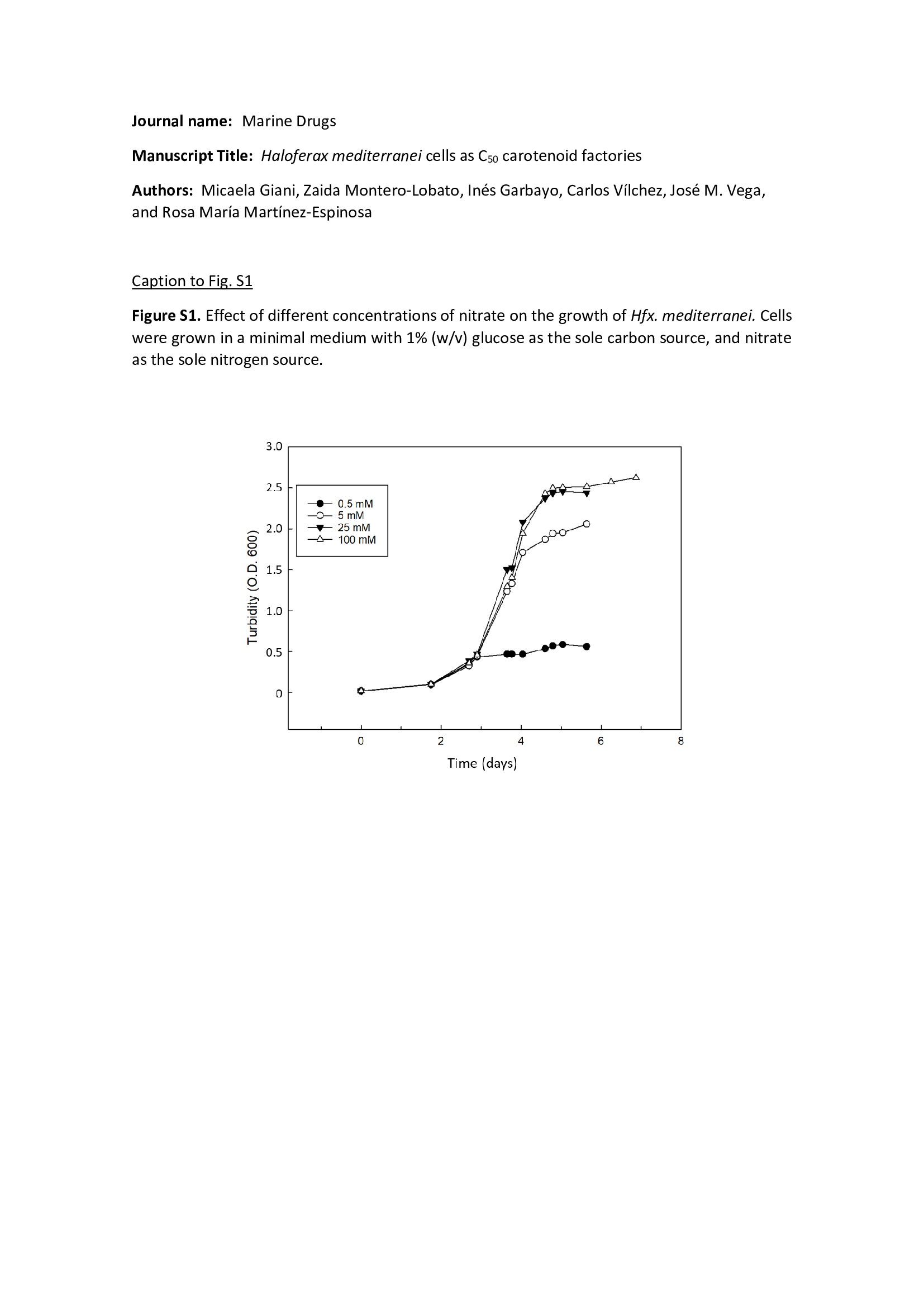

Supplement: Supplementary file 1 [file marinedrugs-19-00100-s001.zip › Fig-S1.jpg]
